# Modified algorithm for managing postoperative osteomyelitis following fracture fixation with Cierny-Mader type

**DOI:** 10.1186/s13018-020-01693-8

**Published:** 2020-06-09

**Authors:** Yanbin Tan, Hang Li, Zhijun Pan, Qiang Zheng

**Affiliations:** Department of Orthopaedic Surgery, the Second Affiliated Hospital, School of Medicine, Zhejiang University, Hangzhou, 310009 China

**Keywords:** Osteomyelitis, Postoperation, Implant

## Abstract

**Background:**

No standardized protocol has been suggested in the treatment of postoperative osteomyelitis following fracture fixation. Our team evaluates the clinical efficacy of the modified algorithm for managing postoperative osteomyelitis following fracture fixation with Cierny–Mader type.

**Methods:**

Ninety-five wounds were reviewed from March 2009 to February 2016 in our hospital. Sixty-one wounds were treated by the modified algorithm as follows: stable hardware + bone not healed Cierny–Mader 1 type = remove hardware, temporary stabilize; stable hardware + bone not healed Cierny–Mader 2 type = retain hardware ; stable hardware + bone not healed Cierny–Mader for type 3 and type 4 = remove hardware, temporary stabilize/Ilizarov technique; unstable hardware + bone not healed = remove hardware, temporary stabilize/Ilizarov technique; and stable hardware + bone healed = remove hardware. Thirty-four wounds were treated by the conventional algorithm. Autodermoplasty, flap transfer, myocutaneous flap, and other methods including antibiotic irrigation and drug delivery system were used in wound repair.

**Results:**

The patients treated with modified algorithm had a significantly reduced recurrence (*P* < 0.01) and increased results of negative bacterial cultures (*P* < 0.01); however, a decrease in the number of retained hardware cases was observed (*P* < 0.05). For those treated with tissue reconstruction, there was no significance (*P* > 0.05) compared with the conventional group.

**Conclusions:**

The modified algorithm for the postoperative osteomyelitis following fracture fixation according to the stability of the hardware and Cierny–Mader type represents a good clinical efficacy in the management of postoperative osteomyelitis. This procedure is simple and shows promising results; more clinical evidence is needed to confirm the existing findings and optimize the treatment of postoperative osteomyelitis following fracture fixation.

## Background

Postoperative osteomyelitis (PPO) following fracture fixation is characterized by infection of the bone and marrow, and most caused by direct or indirect local invasion by microorganisms from adjacent contaminated soft tissue after trauma, during reconstructive bone surgery or insertion of an implant. With the improving of implants techniques, greater numbers of fractures are treated operatively increasing the number of implant -related infections. The incidence of PPO ranges from 5 to 10% in implant associated fractures and extends to approximately 80% of osteomyelitis cases [1].

Radical debridement, fracture stabilization, and adequate soft-tissue coverage are the principal treatment therapy. In particular, the mechanical stability and evaluation of implants is critical to efficacy interpretation for improving outcomes in the treatment of PPO following fracture fixation, tactics based upon clinical evaluation should be focused on the mechanism of the implant; however, there is minimal research progress and few innovations that change the clinical practice and outcomes.

Although there is great variability of strategies for the implant in the treatment of PPO among surgeons with well documented favorable results using negative pressure wound therapy (NPWT) therapy [2, 3], but no standardized protocol has been suggested.

Cierny and Mader developed a detailed classification with osteomyelitis, which applies best to long and large bones. The classification combines four anatomic types (the disease) with three physiologic classes (the host) to define the clinical stages and incorporate the prognostic factors [4]. Class A patients have normal systemic defenses, metabolic capabilities, and vascular supply to the limb. Class B patients have a local (trauma, prior surgery, local inflammation) or systemic (immune suppressed, on corticosteroids, peripheral vascular disease) deficiency. Class C patients are those in whom the treatment of the disease (the infection) is worse than the infection itself. These patients have a poor prognosis for cure [5].

In this article, our team tries to evaluate the clinical efficacy of the tactics for the implant of modified algorithm in comparison to conventional algorithm [6], as an alternative treatment before addressing more aggressive surgical strategies in PPO following fracture fixation with Cierny–Mader type.

## Methods

The authors reviewed a consecutive series of 93 patients between March 2009 and February 2016 in our hospital. There were 60 patients (48 males and 12 females), and 61 wounds (35 tibia, 15 femur, 5 fibula, 2 radius, 2 ulna, and 2 humerus) with an average age of 43.4 years (range, 18–82 years) in modified group. Cultures that performed by direct biopsy from the involved bone at time of debridement, and local symptoms, clinical examination (X-ray, CT, fever, white blood cell count, erythrocyte sedimentation rate, and C-reactive protein concentration) were used to diagnose osteomyelitis. Fifty-three wounds were found to have positive cultures, and 8 wounds of culture-negative. The bacterial species cultured were 17 MRSA, 12 *Staphylococcus aureus*, 6 *Pseudomonas aeruginosa*, 4 *Escherichia coli*, 4 MRSE, 3 *Enterobacter cloacae*, 2 *Acinetobacter baumannii*, and 5 of *Enterococcus faecium*, *Klebsiella*, hemolytic *Streptococcus*, *Staphylococcus epidermidis*, and *Proteus* species. There were 12 wounds that was associated with Cierny–Mader type 1, 10 wounds belong to type 2, 39 wounds belong to types 3 and 4. Fifty-six patients belong to class A, 4 patients belong to class B with systemic factors of 1 patient and diabetes mellitus, 1 patient with steroid therapy, and 2 patients with tobacco abuse.

There were 33 patients (22 males and 11 females) in conventional group, and 34 wounds (26 tibia, 3 femur, 5 of radius, ulna, elbow, humerus, and fibula) with an average patient age of 44.7 years (range, 22–80 years). The bacterial species Culture and clinical examination are same as modified group. Thirty wounds were found to have positive cultures, and 4 cases with negative cultures. Thirty wounds were found to have positive cultures, and 4 wounds of culture-negative. The bacterial species cultured were 16 Staphylococcus aureus, 3 Pseudomonas aeruginosa, 3 Escherichia coli, 2 Klebsiella, 2 MRSE, and 4 with Hemolytic streptococcus, Staphylococcus epidermidis, Acinetobacter, and MRSA. There were 5 wounds that were associated with Cierny–Mader type 1, 6 wounds belong to Cierny–Mader type 2, and 23 wounds for both types 3 and 4. Thirty patients belong to class A, 3 patients for class B with systemic factors of 1 patient who had diabetes mellitus, and 2 patients with tobacco abuse.

The average course of infection in the modified group was 3.6 months (rang, 3 weeks–40 months), the average course of infection in conventional group was 3.3 months (range, 3 weeks–22 months). The NPWT sponges (KCI, TX, USA) were cut to fill and cover the wound after the procedure of surgical debridement, the distal end of the drainage tube connected a vacuum bottle with 20–60 KPa, or a cyclical negative pressure container, and the sponges were changed every 3–4 days.

Antibiotics were started empirically in patients after cultures have been obtained, at the time of debridement. Antibiotic treatment guideline and the antibiotics used were tailored to the recovered bacteria and their susceptibility pattern [7, 8]. In all cases, appropriate antibiotic coverage was gained and maintained for the duration of the treatment protocols [4].

### Surgical procedure

Conventional treatment consisted of re-exploration, removal, and debridement of all necrotic nonviable tissues including free sequestrum. The wound was washed with hydrogen peroxide and rinsed with saline solution. The extent of infection and debridement determined the subsequent treatment. The treatment algorithm for the implant was modified with reference to Ziran [6] (Table 1).

**Table 1:** Treatment algorithm for the implant

Conventional algorithm	Modified algorithm
I) Stable hardware + bone not healed = retain hardware, antibiotics until healed, then hardware removal. II) Unstable hardware + bone not healed = remove hardware, antibiotics, temporary stabilization, spacer, and reconstruction when clean. III) Stable hardware + bone healed = remove hardware, debride with effort not to destabilize, control dead space, and antibiotics. IV) Stable hardware + bone not healed + systemic effects =remove hardware, temporary stabilize, spacer, antibiotics, and reconstruction when able, consider amputation if bad host.	1. Stable hardware + bone not healed Cierny–Mader 1 type = remove hardware, temporary stabilize + antibiotic cement-coated (ACC) rods/Ilizarov technique, debridement, soft-tissue coverage, and reconstruction when clean. 2. Stable hardware + bone not healed Cierny–Mader 2 type = retain hardware, debridement, soft-tissue coverage, bone healed then hardware removal. 3. Stable hardware + bone not healed Cierny–Mader types 3 and 4 = remove hardware, temporary stabilize/Ilizarov technique, debridement, soft-tissue coverage, and reconstruction when clean. 4. Unstable hardware + bone not healed = remove hardware, temporary stabilize/Ilizarov technique, debridement, soft-tissue coverage, and reconstruction when clean. 5. Stable hardware + bone healed = remove hardware, debridement, soft-tissue coverage.

Autodermoplasty, flap transfer, myocutaneous flap, and the other methods including antibiotic irrigation and drug delivery system (DDS) were used in wound repair. The local symptoms, fever, white cell count, erythrocyte sedimentation rate, and C-reactive protein concentration were monitored to evaluate the infection.

### Statistical analysis

Statistical evaluation of the data was carried out using the SPSS Statistics for Windows, version 21.0 (IBM Corp., Armonk, NY, USA). The significant differences between variables were tested using *x*^2^ test. *P* value< 0.05 was considered to be statistically significant.

## Results

**Fig. 1:**
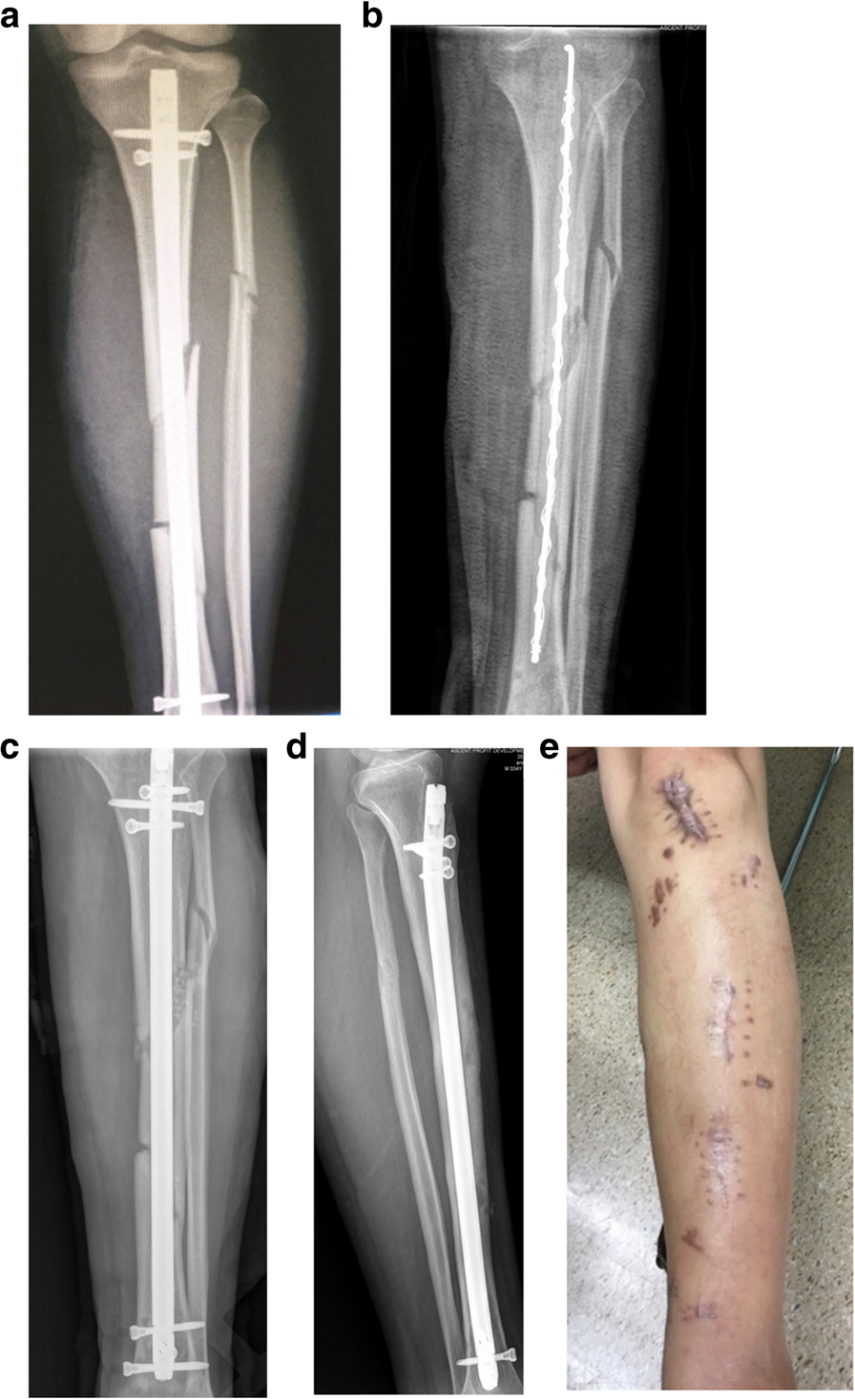
Stable hardware + bone not healed Cierny–Mader 1 type = remove hardware, temporary stabilize + antibiotic cement-coated (ACC) rods, debridement, and reconstruction when clean. **a** Radiographs of primary internal fixation. **b** The tibia locking intramedullary nail was removed and replaced by antibiotic cement-coated (ACC) rods. **c** Reconstruction with locking intramedullary nail with drug delivery system (DDS) when clean. **d** Radiograph 12 months after treatment. **e** After 12 months of treatment, the wound was stable

**Fig. 2:**
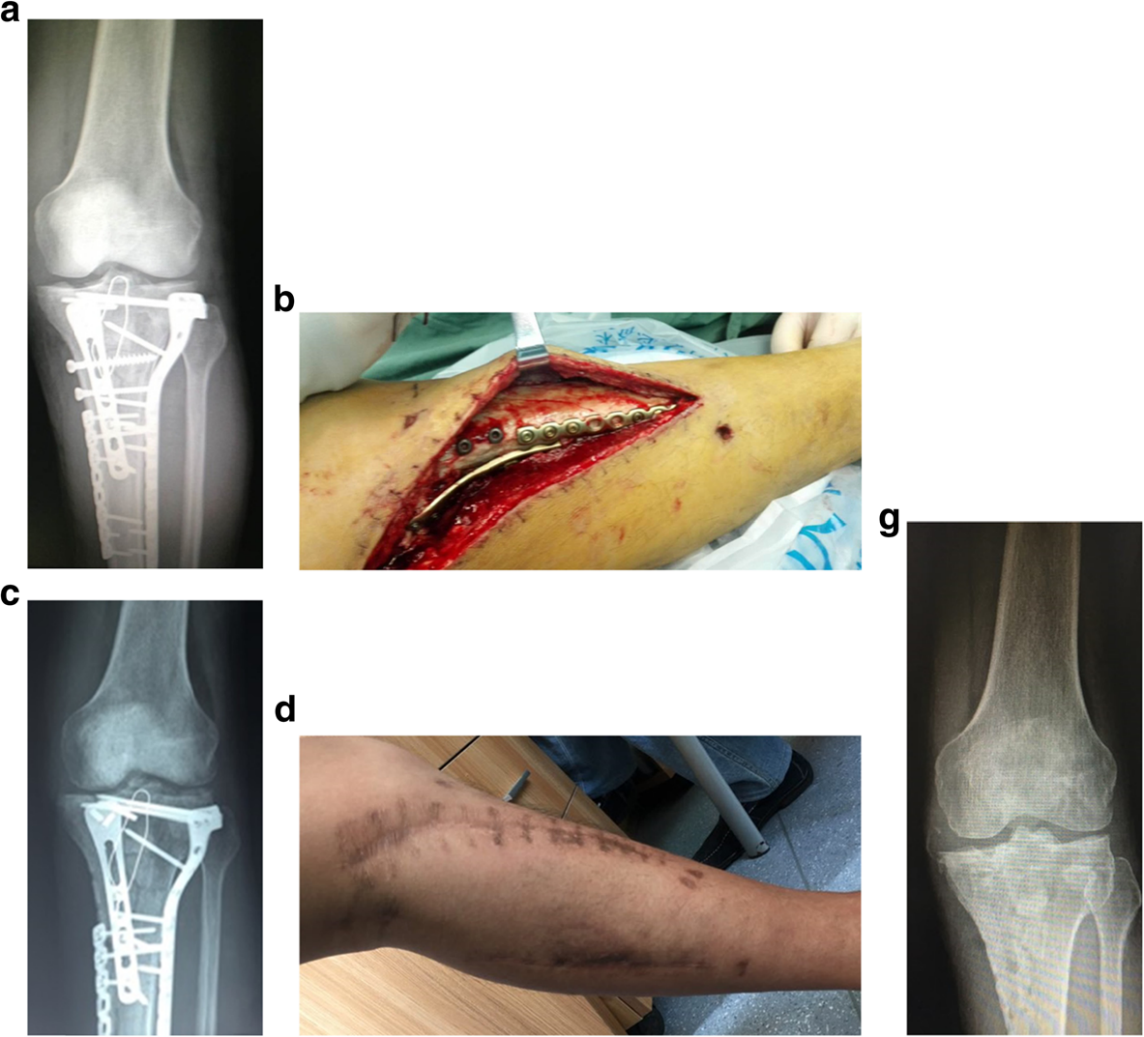
Stable hardware + bone not healed Cierny–Mader 2 type = retain hardware, debridement, soft-tissue coverage, bone healed, then hardware removal. **a** Radiographs of primary internal fixation. **b** Debridement. **c** Radiographs 1 month after treatment. **d**–**f** After 12 months of treatment, the wound was stable. **g** Radiograph 12 months after treatment

**Fig. 3:**
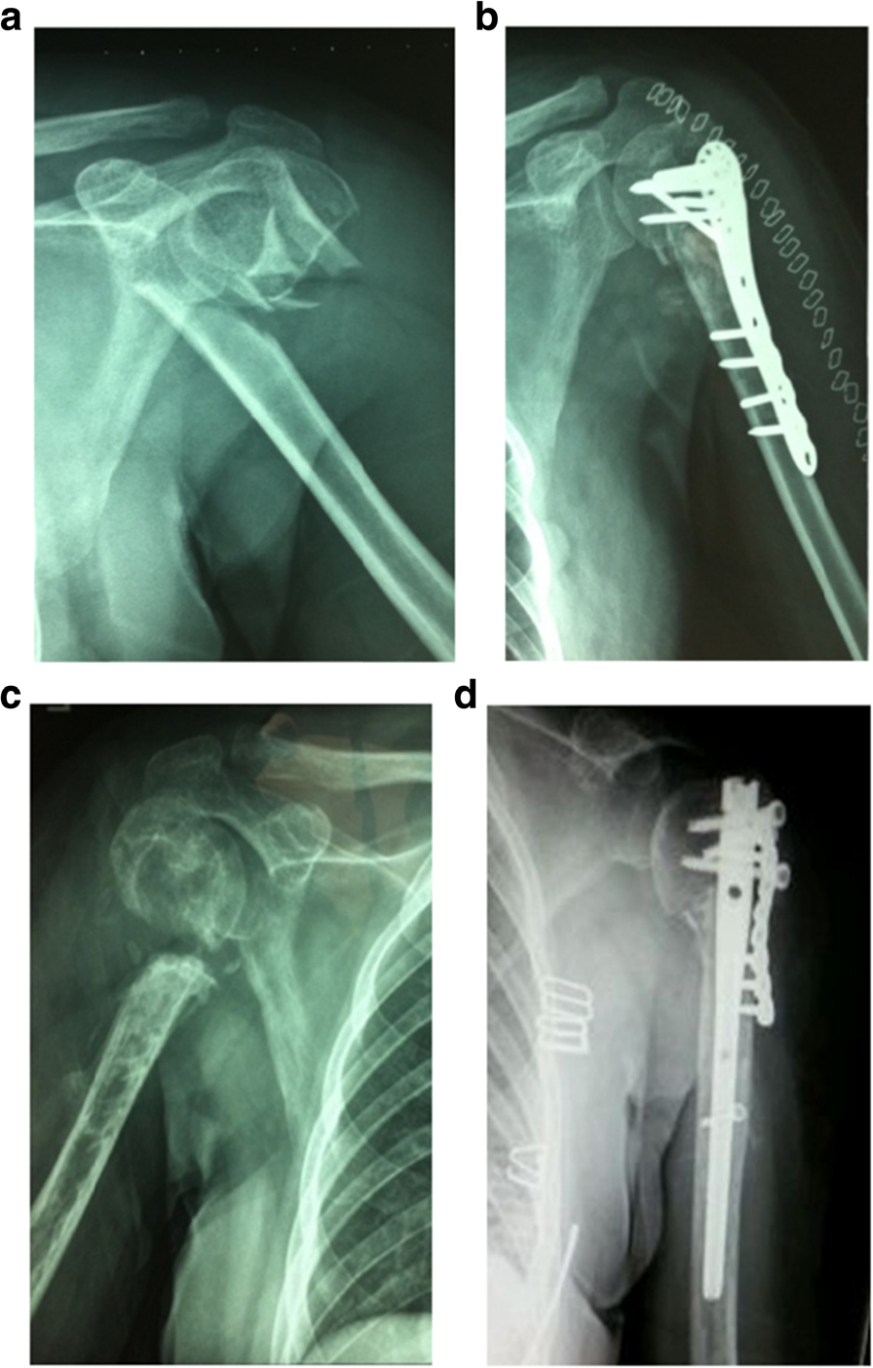
Stable hardware + bone not healed Cierny–Mader types 3 and 4 = remove hardware, temporary stabilize, and reconstruction when clean. **a** Radiograph of humerus fracture. **b** Radiographs of primary internal fixation. **c** The implant was removed with space technique. **d** Reconstruction with locking intramedullary nail and plate when clean

**Fig. 4:**
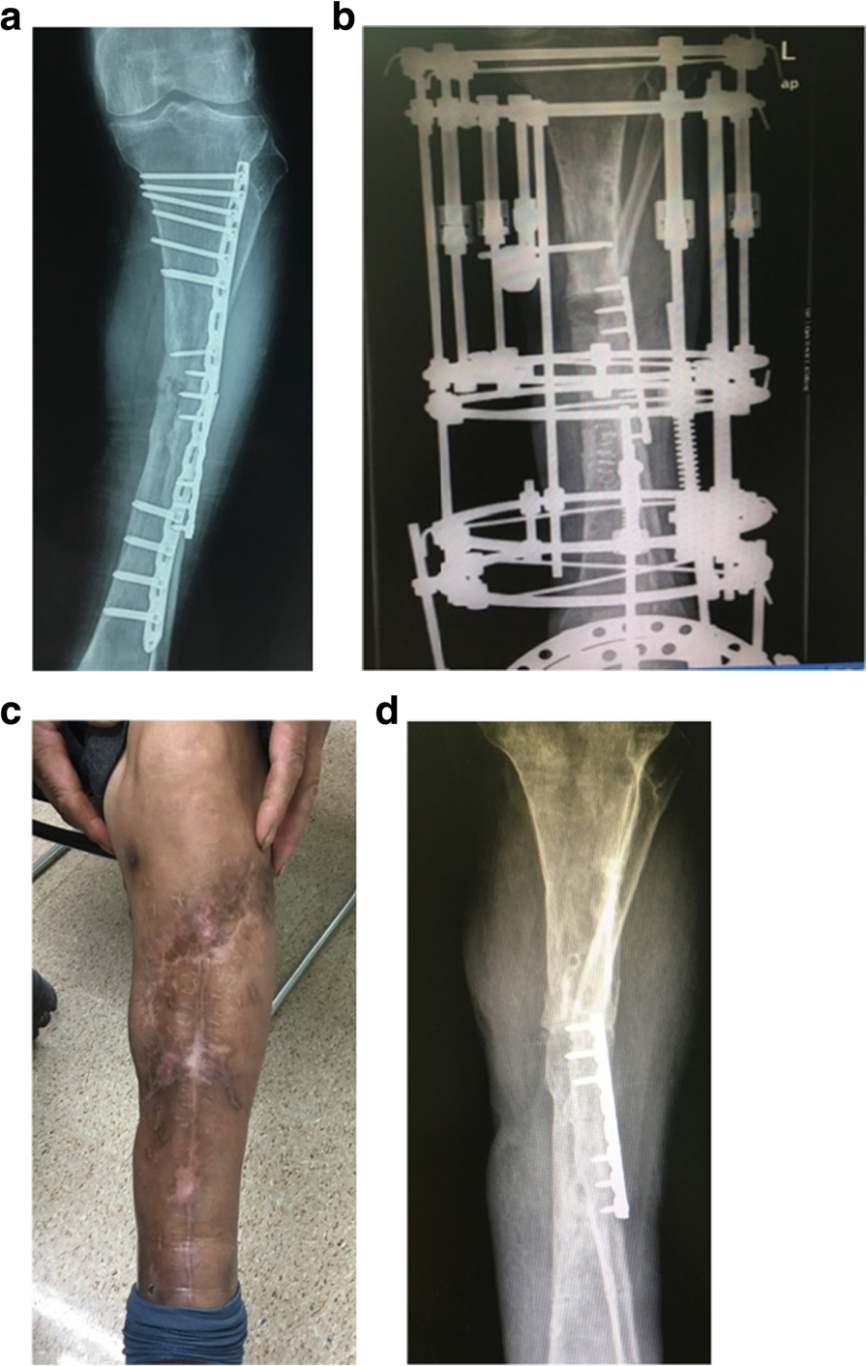
Unstable hardware + bone not healed = remove hardware, temporary stabilize/Ilizarov technique, debridement, soft-tissue coverage, and reconstruction when clean. **a** Radiographs of primary internal fixation. **b** The implant was removed and replaced by Ilizarov external fixation with segmental bone transport technique. **c** After 18 months of treatment, the wound was stable. **d** Radiograph 18 months after treatment

**Fig. 5:**
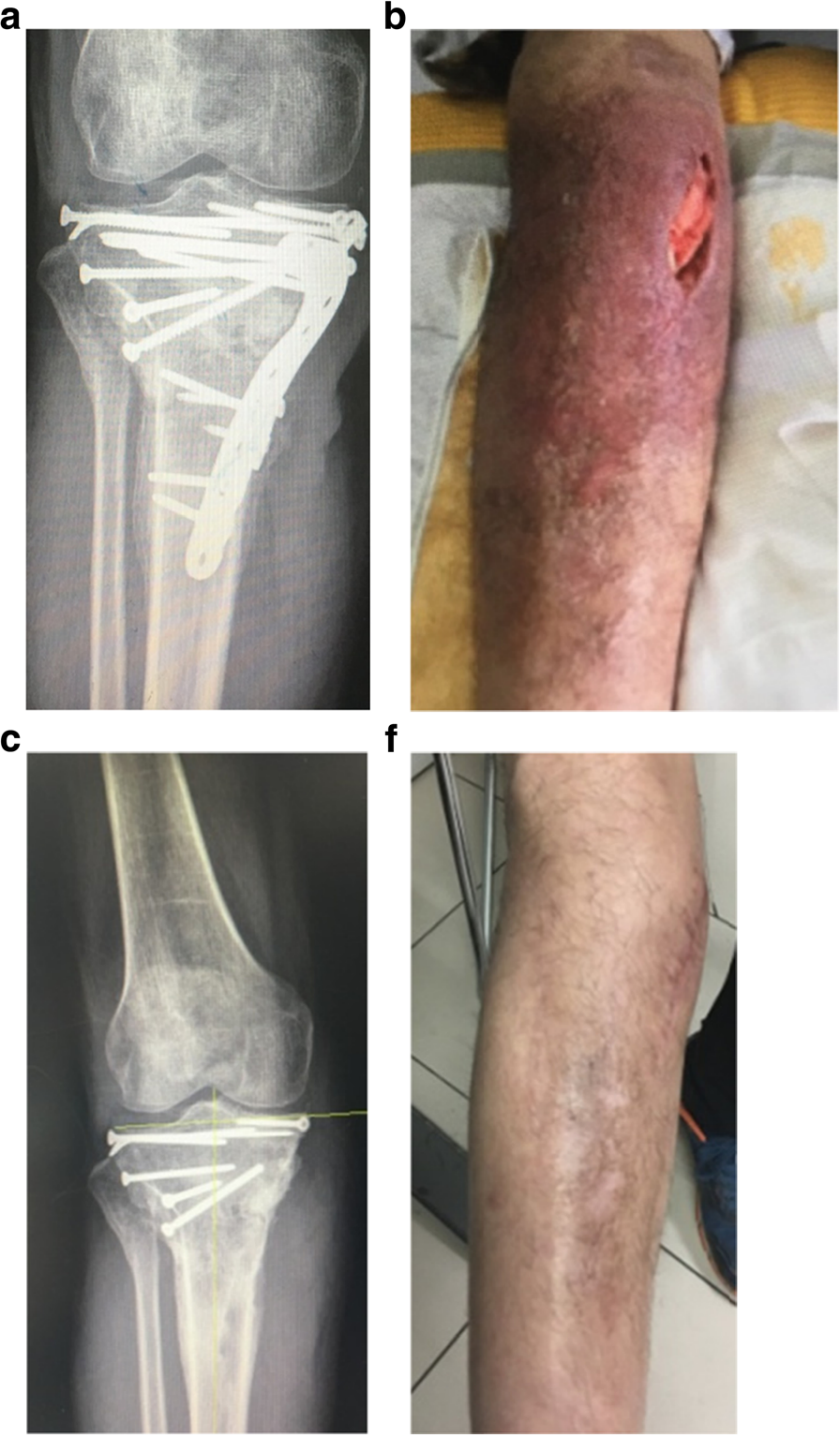
Stable hardware + bone healed = remove hardware, debridement, soft-tissue coverage. **a** Radiographs of primary internal fixation. **b** Partial exposure of the wound. **c** The plate was removed. **d** After 24 months of treatment, the wound was stable

At the modified group, the number of times for debridement averaged 3.4 times (range, 2–5), the bacterial species culture turned out to be negative in 45 wounds (84.9%). There were 8 cases (13.1%) which had hardware retained, 53 cases (86.9%) had hardware removed and 45 cases (84.9%) with temporary stabilize/ Ilizarov technique. 48 wounds (78.7%) required tissue reconstruction, including 10 wounds (16.4%) requiring autodermoplasty, 38 wounds (62.3%) required tissue reconstruction, including 28 (45.9%) myo-cutaneous flap, 10 (16.4%) flap transfer. All patients were followed up at an average of 15 months (range 12–24 months) post-operation coverage (Figs. 1, 2, 3, 4 and 5), and reported only 2 wounds (2 Cierny–Mader type 3 or type 4) of recurrence was found 1 and 2 months respectively.

At conventional group, the number of times for debridement averaged 3.6 times (range, 1–5). The bacterial species culture turned out to be negative in 15 wounds (50%). There were 11 cases (32.3%) which had hardware retained, 23 cases (64.7%) had hardware removed, including 25 (86.2%) with temporary stabilize/Ilizarov technique. Twenty-six wounds (76.5%) required tissue reconstruction, with 21 wounds (61.8%) of autodermoplasty and myo-cutaneous flap, 5 wounds (14.7%) of flap transfer. All patients were followed with an average of 14 months (range, 12–20 months) post coverage, and 7 wounds (4 Cierny–Mader type 1, 3 Cierny–Mader type 3 or type 4) of recurrence were found after 1 month discharge.

When comparing both groups, we found that, the cases of Cierny–Mader type and further tissue reconstruction did not differ significantly (*P* > 0.05), furthermore, there were more hardware retained at conventional group (*P* < 0.05), more wounds turned out to be negative of bacterial species culture at modified group (*P* < 0.01), and the recurrence wounds were significantly decreased at modified group (*P* < 0.01) (Table 2).

**Table 2:** Clinical characteristics of patients with modified and conventional management

Characteristic	Modified	Conventional	Value	*P* value
Male	48	22	2.03^b^	*P > 0.05*
Female	12	11		
Wounds	61	34		
Hardware retained	8	11	5.05^a^	*P* < *0.05*
Cierny–Mader type 1	*12*	*5*	0.37^b^	*P > 0.05*
Cierny–Mader type 2	*10*	*6*		
Cierny–Mader types 3 and 4	*39*	*23*		
Bacterial to be negative	45	15	8.25^a^	*P* < 0.01
Tissue reconstruction	38	26	2.00^b^	*P > 0.05*
Recurrence	2	7	7.63^a^	*P* < 0.01

^a^Statistically significant difference exists between the two groups

^b^Statistical significance does not exists between the two groups

## Discussion

PPO following fracture fixation has the characteristic of exacerbation and remission with successive soft tissue and bones affected [9], and the osteomyelitis could be initiated by bacterial inoculation during trauma or fracture fixation. Improved strategies for management of the osteomyelitis are needed. It has been proved that antibiotic treatment and appropriate surgical tactics is easier to retain an implant with a single infected low-grade organism than one afflicted with highly virulent (i.e., MRSA) or multiple organisms [10]. A much better insight into the role of debriding bacteria infected tissue and improving blood supply are the targets for the development of new surgical techniques.

NPWT technique creates a barrier and a sub-atmospheric pressure to facilitate wound healing, the stretching effect results in increasing new tissues formation, and reducing the need for muscle flaps [11–14], and thus decreases the complexity of the extent of tissue reconstruction ladder [15]. It has been convincingly demonstrated that, osteomyelitis can be eradicated by thorough debridement, may however be difficult for the wound with PPO following fracture fixation. Furthermore, fracture stabilization and adequate soft-tissue coverage is a big problem in this treatment. The current use of local muscle flaps and microvascular free-tissue transfer has made soft-tissue closure, obliteration of dead space, and wound-healing possible in almost all patients. Fortunately, there is the involvement of plastic surgeons in our treatment of soft tissue reconstruction with sophisticated techniques; the extent of tissue damage is not a problem in the treatment.

Cierny and Mader developed a detailed classification for the treatment of osteomyelitis, the various types of osteomyelitis require differing medical, and surgical therapeutic strategies, which were ignored in the conventional algorithm, the modified algorithm for managing postoperative osteomyelitis following fracture fixation were required to improve the standardized protocol.

The management of implants concerning fracture stabilization tactically depends on the osteomyelitis type, in the cases of Cierny–Mader type 2, the implant potentially could be covered gradually by the granulation tissue. The stable plates and screws should be retained [16], but the isolated loosen screws or pin should be removed, and the unstable hardware should be replaced by appropriate fixation such as bridge plate as external fixation. The treatment of Cierny–Mader type 1 is especially difficult, the primary stability of the fracture is very important, and the ACC rods with DDS provide stability as well as delivering antibiotics [17]. The tibia is the most frequent site of an open fracture and the most common site of PPO following fracture fixation [6, 7]. Although the infection rate after open reduction and internal fixation of closed fractures is significantly lower [8], the incidence of osteomyelitis after open fractures is reported to be 2 to 16%. However, the incidence of infection in the tibia is reported to be as high as 23% depending significantly on the grade of trauma and the type of treatment administered, there were totally 61 tibia wounds (64.2%) in our study, most were classified as either Cierny–Mader type 3 or type 4. The primary closure of the tissue defect and the bone healing is challenging, external fixation is a better choice to facilitate the treatment, the Ilizarov frame could be used to treat the bone defect, and the wound could be gradually closed with less complications.

In our study, the modified algorithm represents a good clinical efficacy in treating PPO following fracture fixation, the wound bacterial species culture turned out to be negative with the significance differences between the conventional group (*P* < 0.01), and the recurrence cases were significantly decreased (*P* < 0.01), but more hardware were retained at the conventional group (*P* < 0.05), we explained it using the classification of “hardware stable + bone not healed,” less hardwares were retained at the modified group. Furthermore, the modified algorithm is only as a guide and baseline along with which to individualize the specific patient’s treatment. This article outlines the algorithm for managing PPO following fracture fixation that aids the surgeon in decision-making and provides the surgeon with basis in the restoration of the extremity. More clinical studies are required to confirm the modified algorithm.

## Conclusion

We summarize and modified the conventional treatment algorithm according to Cierny–Mader type for the treatment of PPO following fracture fixation with the clinical experiments. This procedure is simple and shows promising results; however, more clinical evidence is needed to confirm the existing findings and optimize the treatment of PPO following fracture fixation.

## Data Availability

The datasets generated and/or analyzed during the current study are available from the corresponding author by reasonable request.

## Abbreviations

PPO: Postoperative osteomyelitis
NPWT: Negative pressure wound therapy
ACC: Antibiotic cement-coated
DDS: Drug delivery system
